# Plant-derived compounds normalize platelet bioenergetics and function in hyperglycemia

**DOI:** 10.1016/j.rpth.2024.102548

**Published:** 2024-08-14

**Authors:** Julia S. Gauer, Abigail Ajanel, Lutale M. Kaselampao, Isabel Candir, Amanda D.V. MacCannell, Lee D. Roberts, Robert A. Campbell, Robert A.S. Ariëns

**Affiliations:** 1 Discovery and Translational Science Department, Leeds Institute of Cardiovascular and Metabolic Medicine, University of Leeds, Leeds, United Kingdom; 2 Molecular Medicine Program, University of Utah, Salt Lake City, UT 84112, USA; 3 Department of Pathology, University of Utah, Salt Lake City, UT 84112, USA; 4 Department of Internal Medicine, University of Utah, Salt Lake City, UT 84112, USA; 5 Department of Emergency, Washington University, Saint Louis, MO 63110, USA

**Keywords:** bioenergetics, clot structure, diabetes, hyperglycemia, oxidative stress, plant-derived, platelets, polyphenols

## Abstract

**Background:**

Polyphenols have been shown to decrease oxidative stress and modulate glycemic response. Nevertheless, their effect on platelet bioenergetics and clot structure in diabetes and hyperglycemia is unknown.

**Objectives:**

To investigate the effect of polyphenols on human platelet bioenergetics and its subsequent effect on clot structure in normoglycemia vs acute hyperglycemia *in vitro*.

**Methods:**

Four polyphenols (resveratrol, hesperetin, epigallocatechin gallate [EGCG], and quercetin) were selected for initial analysis. Healthy volunteers’ isolated platelets/platelet-rich plasma were treated with 5 or 25 mM glucose to represent normoglycemia and acute hyperglycemia, respectively. Platelet-derived reactive oxygen species (ROS), citrate synthase activity (mitochondrial density), mitochondrial calcium flux, and mitochondrial respiration were performed following exposure to polyphenols (20 µM, 1 hour) to determine their effects on platelet bioenergetics. Procoagulant platelets (annexin V) and fibrin fiber density (Alexa Fluor-488 fibrinogen; Invitrogen) were analyzed by laser scanning confocal microscopy, while clot porosity was determined using platelet-rich plasma following exposure to polyphenols (20 µM, 20 minutes).

**Results:**

Acute hyperglycemia increased ROS, mitochondrial calcium flux, maximal respiration, and procoagulant platelet number. Resveratrol, quercetin, and EGCG reduced platelet ROS in normoglycemic and acute hyperglycemic conditions. Mitochondrial density was decreased by quercetin and EGCG in normoglycemia. Resveratrol and EGCG reduced mitochondrial calcium flux in acute hyperglycemia. Resveratrol also decreased procoagulant platelet number and attenuated oxygen consumption rate in normoglycemia and acute hyperglycemia. No effect of hyperglycemia or polyphenols was observed on fibrin fiber density or clot pore size.

**Conclusion:**

Our results suggest polyphenols attenuate increased platelet activity stemming from hyperglycemia and may benefit thrombosis-preventative strategies in patients with diabetes.

## Essentials

• Altered platelet activity and coagulation are associated with diabetes and thrombosis risk.
• Polyphenols have known antioxidant properties and can modulate glycemic response.
• Polyphenols attenuated increased platelet bioenergetics stemming from acute hyperglycemia.
• Compounds may have benefits in thrombosis prevention strategies for patients with diabetes.

## 1 Introduction

Platelets and fibrin are key players involved in hemostasis [1]. Elevated fibrin formation and altered platelet function can lead to increased risk of pathological thrombosis [2]. Thromboembolic disorders are a major health burden and cause of death worldwide [3,4]. Diabetes mellitus is another key public health concern, expected to affect 698 million adults by 2045 without implementation of effective preventative measures [5]. Despite advances in therapy and prevention strategies, up to 65% of deaths in individuals with diabetes are attributed to thrombosis [6,7]. Thrombosis and diabetes risk are intrinsically linked, highlighting the need for further investigations on novel ways to prevent and lessen thrombosis events in patients with diabetes.

Platelet bioenergetics is critical to the production and expenditure of substrates that support major processes such as activation, adhesion, and aggregation [8]. Altered platelet activity and coagulation have been associated with diabetes [[9], [10], [11]]. To add to that, abnormal clot structure, presented as denser and less porous with increased resistance to fibrinolysis, has been described in patients with diabetes [12,13]. Platelet mitochondrial respiration and energy metabolism have been associated with development of procoagulant platelets, a subpopulation of activated platelets that contribute to increased coagulation [[14], [15], [16], [17], [18], [19]]. The effects of hyperglycemia on platelet bioenergetics, contributing to the development of procoagulant platelets, and its impact on clot structure remain to be described.

Dietary habits influence risk of cardiovascular disease and diabetes [20]. Diets rich in fruits, vegetables, and whole grains, for instance, have been shown to contribute to management and prevention of type 2 diabetes by improving glycemic control [21]. Polyphenols are bioactive plant-derived compounds with well-characterized antioxidant properties and reported cardiovascular and cardiometabolic benefits [[22], [23], [24]]. Polyphenols have been shown to modulate postprandial glycemic response *in vivo* [25,26] and diminish oxidative stress induced by hyperglycemia [27]. Nevertheless, the effect of polyphenols on platelet bioenergetics and its subsequent contribution to changes in clot structure in hyperglycemia remain to be established.

In this study, we determined the effect of acute hyperglycemia and polyphenols on platelet bioenergetics and clot structure. We found that polyphenols rescue perturbed platelet bioenergetics induced by hyperglycemia.

## 2 Methods

### 2.1 Plant-derived compounds

Four polyphenols, namely, resveratrol (M02442, Fluorochem), quercetin (N1841, Stratech Scientific), epigallocatechin gallate (EGCG; A2600, Stratech Scientific), and hesperetin (H4125, Merck) were reconstituted in dimethyl sulfoxide (DMSO; D5879, Honeywell) to 20 to 44 mM and stored at 4 °C or −20 °C (EGCG). The flavonoids quercetin, EGCG, and hesperetin were chosen based on previous studies demonstrating their effect on sugar uptake *in vitro* and postprandial glycemia modulation *in vivo* [25,[27], [28], [29], [30]]. Resveratrol was included based on previously shown effects on platelet aggregation and thrombus formation in diabetes [31,32]. Polyphenols were diluted from stock concentrations in saline to a final concentration of 20 μM (<0.05% DMSO). Stock DMSO was diluted accordingly (based on 44 mM) and used in experiments throughout as a control. Chosen concentrations were consistent with a previous study on oxidative stress in hyperglycemia [27]. Table 1 summarizes the characteristics of each compound included in this study from the relevant literature.

**Table 1:** Summary of compound characteristics and relevant previously reported effects.

Compound	Classification	Formula	Molecular mass (g/mol)	Structure	Common source	Relevant effects
Resveratrol	Stilbene	C14H12O3	228.25		Grape	↓ aggregation ↓ collagen-induced thrombus formation in diabetes [31,32]
Quercetin	Flavonoid	C15H10O7	302.23		Apple	↓ ROS in normoglycemia and hyperglycemia (HepG2 cells) ↓ sugar transport *in vitro* [27,29]
Hesperetin	Flavonoid	C16H14O6	302.28		Citrus fruit	↓ sugar transport *in vitro* Modulated postprandial glycemic response [25,29]
EGCG	Flavonoid	C22H18O11	458.37		Green tea	↓ sugar transport *in vitro* ↓ postprandial glycemic response [[28], [29], [30]]

“↓” indicates significant decrease.

ATP, adenosine triphosphate; EGCG, epigallocatechin gallate; ROS, reactive oxygen species.

### 2.2 Human blood collection

Blood samples were collected from the antecubital vein of healthy volunteers in acid citrate dextrose (ACD)-A (platelet isolation) or 0.109 M sodium citrate vacutainers (clot structure experiments). Informed written consent from volunteers was obtained, according to the Declaration of Helsinki. Ethical approval was granted by the University of Leeds Medicine and Health Faculty Research and University of Utah Medical School Ethics Committees. Whole blood was centrifuged within 2 hours of collection at 100 × *g* for 20 minutes without brakes to obtain platelet-rich plasma (PRP).

### 2.3 Reactive oxygen species

Platelet isolation was performed using modified Tyrode’s buffer containing 5.6 mM or 25 mM glucose to represent normoglycemia and acute hyperglycemia, respectively. Platelet activation inhibitor prostaglandin I2 was added to PRP (200 nM) prior to centrifugation at 1000 × *g* for 10 minutes (no brakes). The platelet pellet was washed with modified Tyrode’s buffer, centrifuged (as described previously), resuspended (2 × 10^8^) in warm modified Tyrode’s buffer, and rested at 37 °C for 30 minutes prior to use. Washed platelets were incubated with polyphenols (20 µM) for 1 hour prior to cellular reactive oxygen species (ROS) measurements, quantified by fluorescence intensity of permeant reagent 2′,7′-dichlorofluorescein diacetate (assay kit, 601520, Cayman) at 500/550 nm excitation/emission using a Synergy H1 plate reader (BioTek).

### 2.4 Citrate synthase activity

Platelet isolation and incubation with polyphenols were performed as described above. Platelets were lysed using immunoprecipitation lysis buffer (1:1), and citrate synthase activity, the most commonly used marker of mitochondrial content [[33], [34], [35], [36]], was measured as previously described [33]. Briefly, a reaction mixture composed of 100 mM Tris-HCl pH 8.0, 0.2 mM acetyl coenzyme A (A2056, Merck), 0.1 mM 5,5′-dithiobis (2-nitrobenzoic acid; 22582, Thermo Fisher Scientific), and 1 mM oxaloacetate (omitted in negative controls; 328-42-7, Merck) was added to platelet lysates, in triplicates. Citrate synthase activity was measured at 412 nm every 20 seconds for 10 minutes at 37 °C using a PowerWave microtiter-plate reader (BioTek). Activity represents conversion of substrate (micromoles) into product over time (minutes).

### 2.5 Mitochondrial calcium flux

Platelet isolation and subsequent dilution were performed using Tyrode’s buffer containing 5.6 mM or 25 mM glucose to represent normoglycemia and hyperglycemia, respectively. Platelet activation inhibitor prostaglandin E1 was added to PRP (200 nM) prior to centrifugation at 1000 × *g* for 20 minutes (no brakes). The platelet pellet was washed with Tyrode’s buffer, centrifuged (as described previously), and resuspended (2 × 10^8^) in warm Tyrode’s buffer. Platelets were labeled with X-Rhod-1 (1 µM; X14210, Invitrogen) calcium indicator dye for 1 hour at 37 °C away from light. Samples were centrifuged at 1000 × *g* for 10 minutes (no brakes), following the addition of prostaglandin E1, resuspended, and diluted to 2 × 10^7^ in Tyrode’s buffer. Samples were incubated with polyphenols (20 µM) for 1 hour at 37 °C away from light. Platelet marker CD41-APC (559777, BD) was added to samples, and mitochondrial calcium flux was measured kinetically with CytoFLEX S Flow Cytometer (Beckman Coulter). Baseline (BL) signal was obtained for 1 minute prior to activation with 100 ng/mL convulxin and 24 mM CaCl_2_. Kinetic profile was obtained using FlowJo software (version 10.9.0; FlowJo, LLC).

### 2.6 Oxygen consumption rate

Platelet isolation was performed as described above and platelets were resuspended in XDMEM media (102353-100, Agilent) supplement with 1 µM pyruvate, 4 mM L-glutamine, and either 5.5 mM or 25 mM D-glucose. Platelets were incubated with polyphenols (20 µM) for 1 hour at 37 °C prior to mitochondrial stress test, performed using a Seahorse XF Analyzer (Agilent) to determine oxygen consumption rate (OCR). Once BL OCR levels were determined (4 measurements of 3 × 3 minutes of mix and read), platelets were activated with 100 ng/mL convulxin, followed by subsequent injections of oligomycin [adenosine triphosphate (ATP) synthase inhibitor; 1 µM], carbonylcyanide-p-trifluoromethoxyphenylhydrazone (uncoupling agent; 0.6 µM), rotenone, and antimycin A (complex I and III inhibitors; 1 µM) to determine OCR profile.

### 2.7 Laser scanning confocal microscopy

Sample preparation and imaging were performed as previously described [37]. Briefly, PRP was diluted 1:6 with saline (154 mM NaCl) containing 0 mM, 5 mM (representative of normoglycemia), or 25 mM glucose (representative of hyperglycemia). Annexin V (1:8; R37176, Invitrogen) and polyphenols (20 µM) were added to samples, which were incubated for 20 minutes at room temperature away from light before spiking with 50 µg/mL Alexa Fluor 488-fibrinogen (F13191, Invitrogen). Tissue factor (1.2 pM; 86196, Stago) and CaCl_2_ (5 mM) were added to the samples to initiate clotting, followed by immediate transfer to Ibidi uncoated m-Slide 0.4 µm (Ibidi GmbH) and placement in a dark humidity chamber for 2 hours. Imaging was performed using a Zeiss LSM800 inverted microscope (Carl Zeiss AG) with a 40× oil immersion lens. Compiled and flattened optical z-stacks (45 × 0.7 µm) were used for fiber density (average fibers from 3 separate locations on the clot crossing 160 µm arbitrary line) and procoagulant platelet number (particle count) measurements on ImageJ (version 2.0, National Institutes of Health).

### 2.8 Platelet deposition under arterial shear flow

Platelets were isolated as described above and resuspended in 4-(2-hydroxyethyl)-1-piperazineethanesulfonic acid (HEPES) Tyrode’s buffer pH 7.4 (128 mM NaCl, 2.8 mM KCl, 20 mM HEPES, 0.4 mM NaH_2_PO_4_, 12 mM NaHCO_3_, 1 mM MgCl_2_, 5 mM glucose, and 0.35% bovine serum albumin) at 6 × 10^8^ platelets/mL. After removing PRP, red blood cells (RBCs) were resuspended in HEPES Tyrode’s buffer pH 6.5 and centrifuged at 200 × *g* for 7 minutes. A second wash was then performed at 1200 × *g* for 7 minutes. Platelets were incubated for 15 minutes with either 20 μM resveratrol (Tocris) or buffer and stained for 15 minutes with 0.5 mM DIOC6(3) (Thermo Fisher Scientific) to label the platelets. Stained platelets were mixed with washed RBCs to reach a 40% hematocrit. A Cellix Mirus Evo Pump 8-channel pulse-free microfluidic syringe pump was used to perfuse the RBC-platelet mixture on a multichannel chip (Vena8 Fluoro+ Biochips, Cellix). The chip was coated with 20 μg/mL of collagen (Chronolog) overnight at 4 °C and then washed and blocked with 1% bovine serum albumin in phosphate-buffered saline (pH 7.4) for 1 hour at 37 °C. The RBC-platelet mixture was then perfused for 5 minutes at 1500 s^−1^. For sample analysis, one section of the channel was selected and recorded for 5 minutes. An image at the 5-minute time point from when platelets first adhered to collagen strands was selected for analysis. Images were analyzed using ImageJ by using an automatic threshold analysis to determine the number of adherent platelets. The number of pixels identified as platelets per field of area was used to calculate platelet adhesion.

Methods relevant to supplementary figures are included as Supplementary Methods.

### 2.9 Statistical analysis

Data are presented as the mean ± SEM. The “n” number represents the number of independent individual repeats. Technical repeats, where relevant, are averaged and account for 1 repeat. Normal distribution of data was assessed with the Shapiro–Wilk test for normality. Differences between conditions were determined by one-way analysis of variance or Kruskal–Wallis test followed by Tukey–Kramer or Dunn–Bonferroni post hoc test to determine significance. Two-way analysis of variance was used to determine differences in OCR. Differences between normoglycemic and hyperglycemic conditions were determined by paired 2-tailed homoscedastic Student’s *t*-test. *P* values <.05 were considered to indicate statistical significance. Data analysis was performed using GraphPad Prism software (version 10.1.1; GraphPad, LLC).

## 3 RESULTS

### 3.1 Polyphenols attenuate platelet cellular ROS

**Figure 1:**
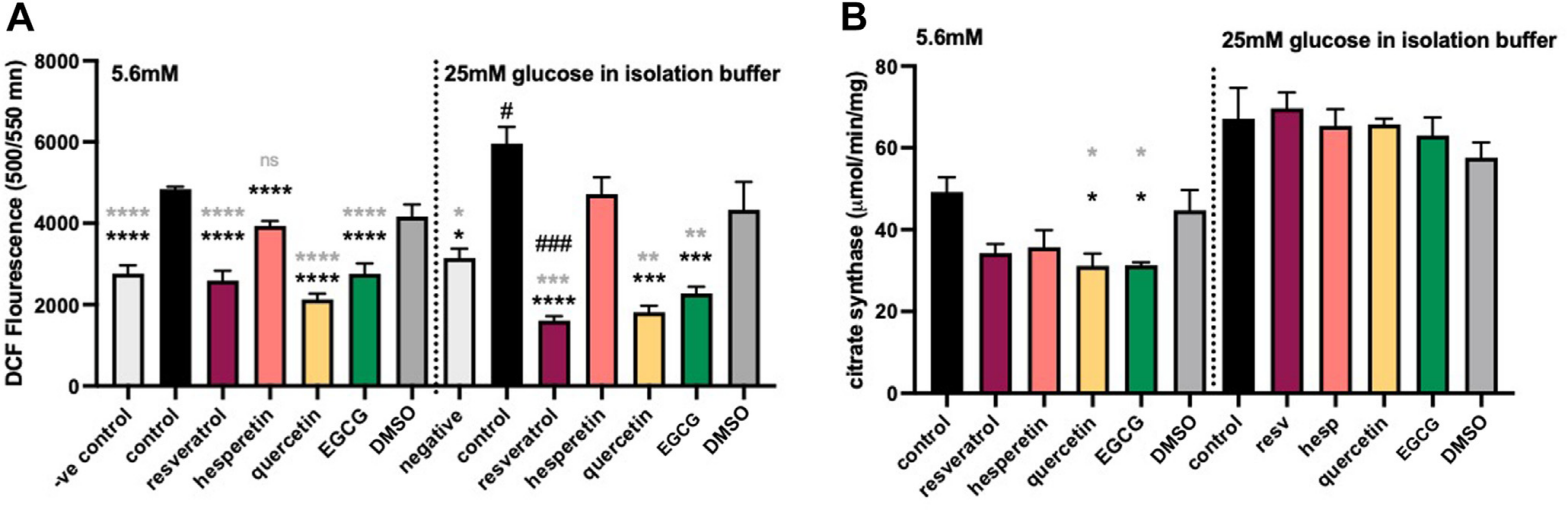
Quantification of platelet-derived reactive oxygen species and platelet mitochondrial density in the presence of polyphenols. Comparison of (A) platelet cellular reactive oxygen species (quantified by fluorescence intensity of permeant reagent 2′,7′-dichlorofluorescein (DCF) diacetate at 500/550 nm excitation/emission;) and (B) platelet mitochondrial density (determined using a citrate synthase activity) in normoglycemic vs hyperglycemic conditions (5.6 mM vs 25 mM glucose in isolation buffer, respectively). Washed platelets were incubated with polyphenols (20 µM) for 1 hour prior to measurements. “−ve control” in A refers to N-acetyl cysteine (provided with a commercial assay kit). Results are shown as mean ± SEM, *n* = 4 with 3 technical repeats. EGCG, epigallocatechin gallate; hesp, hesperetin ns, nonsignificant; resv, resveratrol. ∗*P* < .05; ∗∗*P* < .01; ∗∗∗*P* < .001; ∗∗∗∗*P* < .0001 difference from control (washed platelets only) or dimethyl sulfoxide (DMSO) in black or gray, respectively. ^#^*P* < .01; ^###^*P* < .001 difference from the corresponding condition at 5.6 mM.

Whole platelet ROS, indicative of oxidative stress, and mitochondria-derived ROS were significantly increased (19% ± 1%; *P* < .05) in healthy volunteers’ platelets isolated under acute hyperglycemic conditions (25 mM glucose in isolation buffer) compared with normoglycemic control (Figure 1A; Supplementary Figure S1). Treatment of platelets with polyphenols led to a reduction in total platelet ROS under normoglycemic (46% ± 5%, 56% ± 3%, and 43% ± 5% for resveratrol, quercetin, and EGCG, respectively; *P* < .0001) and hyperglycemic conditions (73% ± 2%, 69% ± 4%, and 62% ± 3% for resveratrol, quercetin, and EGCG, respectively, *P* < .001; Figure 1A). There was no change observed by the compounds on mitochondria-derived ROS (Supplementary Figure S1). As the polyphenols were solubilized in DMSO, the effect of equivalent concentrations of this solvent was also investigated, with no differences in ROS observed. Interestingly, the effect of resveratrol on modulation of whole platelet ROS was more pronounced (38% ± 4%; *P* < .001) under hyperglycemic conditions compared with the decrease observed in the presence of this compound in normoglycemia (Figure 1A).

### 3.2 Polyphenols decrease platelet mitochondrial density

Mitochondria are central regulators of ROS formation; therefore, we investigated whether acute hyperglycemia and/or polyphenols affected mitochondrial density and citric acid cycle activity. Platelet mitochondrial density was determined using the citrate synthase activity assay. There was no significant difference in mitochondrial density in healthy volunteers’ platelets isolated under normoglycemic vs acute hyperglycemic conditions (Figure 1B). Under normoglycemia, quercetin and EGCG led to a significant decrease (31 ± 6 and 31 ± 2, respectively; *P* < .05) in citrate synthase activity (Figure 1B). No significant differences were observed for polyphenols in hyperglycemic conditions or by DMSO control under either condition (Figure 1B).

### 3.3 Acute hyperglycemia increases platelet mitochondrial calcium flux

**Figure 2:**
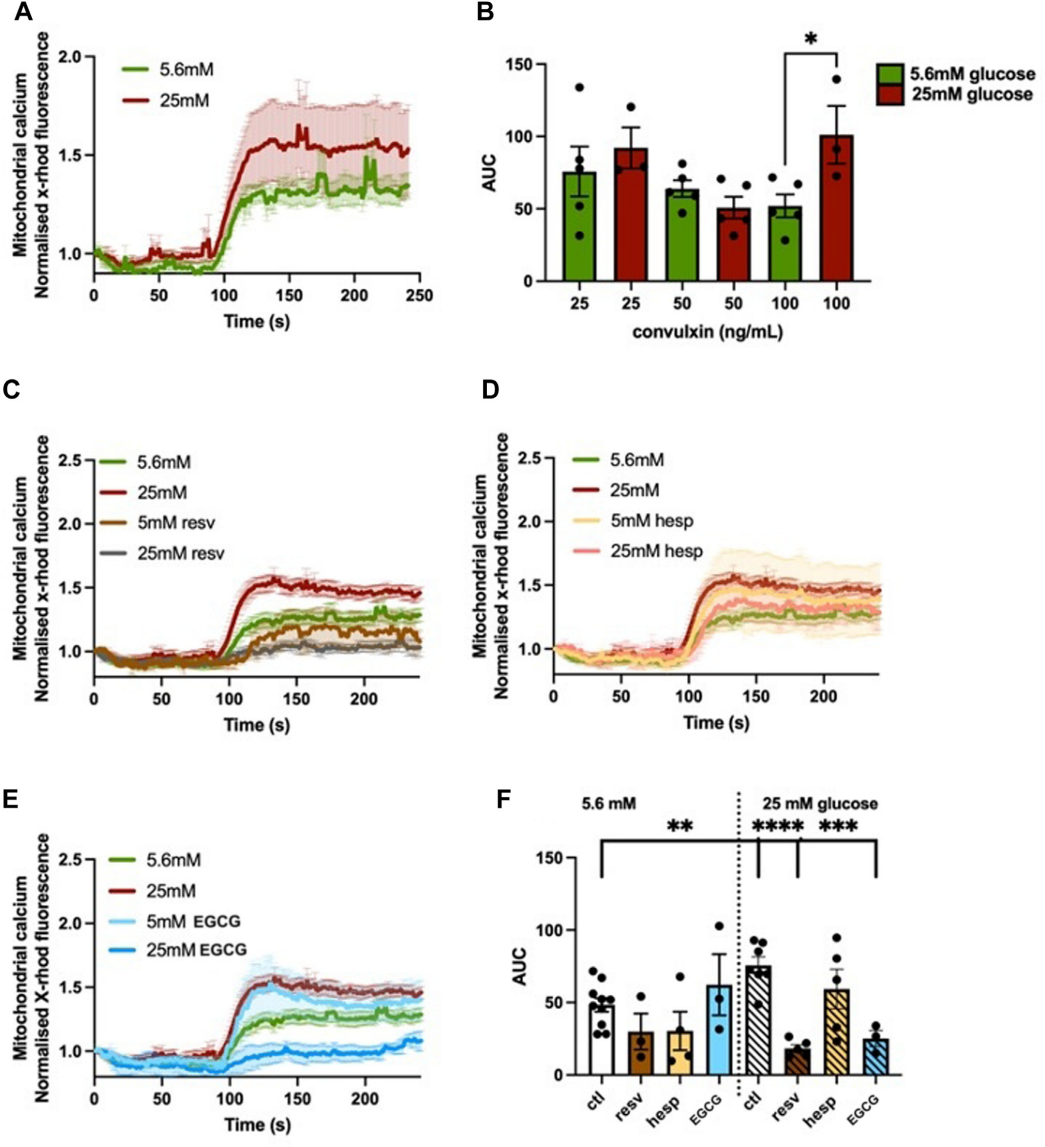
Mitochondrial calcium flux in normoglycemic vs acute hyperglycemic conditions in the absence and presence of polyphenols. Healthy volunteers’ platelets were isolated in isolation media containing 5.6 mM vs 25 mM glucose in isolation buffer (representing normoglycemia and acute hyperglycemia, respectively). Mitochondrial calcium flux was measured kinetically by flow cytometry in platelets labeled with X-Rhod-1 calcium indicator dye for 1 hour activated with convulxin. (A) Different concentrations (25-100 ng/mL) of convulxin were tested, with 100 ng/mL chosen for future experiments based on changes in area under the curve (AUC). (B) Kinetic time series, obtained with FlowJo, for platelets activated with 100 ng/mL convulxin. Following incubation with X-Rhod-1 calcium indicator dye for 1 hour, platelets were incubated with 20 µM (C) resveratrol (resv), (D) hesperetin (hesp), or (E) epigallocatechin gallate (EGCG) for 1 hour prior to analysis. An equivalent concentration of dimethyl sulfoxide in samples with polyphenols was added to controls (ctl). (F) AUC was determined in normoglycemia vs acute hyperglycemia in the presence of polyphenols. Results are shown as mean ± SEM, (A and B) *n* = 3 to 5 and (C–F) *n* = 3. ∗*P* < .05; ∗∗*P* < .01; ∗∗∗*P* < .001; ∗∗∗∗*P* < .0001 difference from ctl.

Mitochondrial calcium flux is critical for cellular function by contributing to the regulation of energy production/expenditure. Platelet mitochondrial calcium flux was determined kinetically by flow cytometry in healthy volunteers’ platelets isolated under normoglycemic vs acute hyperglycemic conditions and stained with calcium indicator dye X-Rhod-1. Different concentrations of agonists were used during method optimization (Figure 2A), with 100 ng/mL convulxin being chosen for further experiments. Under the chosen conditions, there was a significant increase in mitochondrial calcium flux (measured by the area under the curve; 48% ± 8%; *P* < .05) in platelets isolated under acute hyperglycemic conditions compared with normoglycemia control (Figure 2A, B).

### 3.4 Polyphenols attenuate hyperglycemia-induced platelet mitochondrial calcium flux

Once we determined that acute hyperglycemia increased mitochondrial calcium flux, we explored the potential modulation effects of polyphenols. Following platelet isolation and staining with a calcium indicator, platelets were treated with resveratrol (Figure 2C), hesperetin (Figure 2D), or EGCG (Figure 2E) under normoglycemic and hyperglycemic conditions. An equivalent concentration of DMSO in samples with polyphenols was added to control samples without polyphenol compounds. Increased mitochondrial calcium flux area under the curve in acute hyperglycemia (36% ± 7% compared with normoglycemia control with DMSO; *P* < .01) was attenuated following incubation with resveratrol (75% ± 3%; *P* < .0001), and EGCG (67% ± 7%; *P* < .001; Figure 2F). No significant changes were observed under normoglycemia or in the presence of hesperetin under either condition (Figure 2F).

### 3.5 Resveratrol attenuates platelet OCR

**Figure 3:**
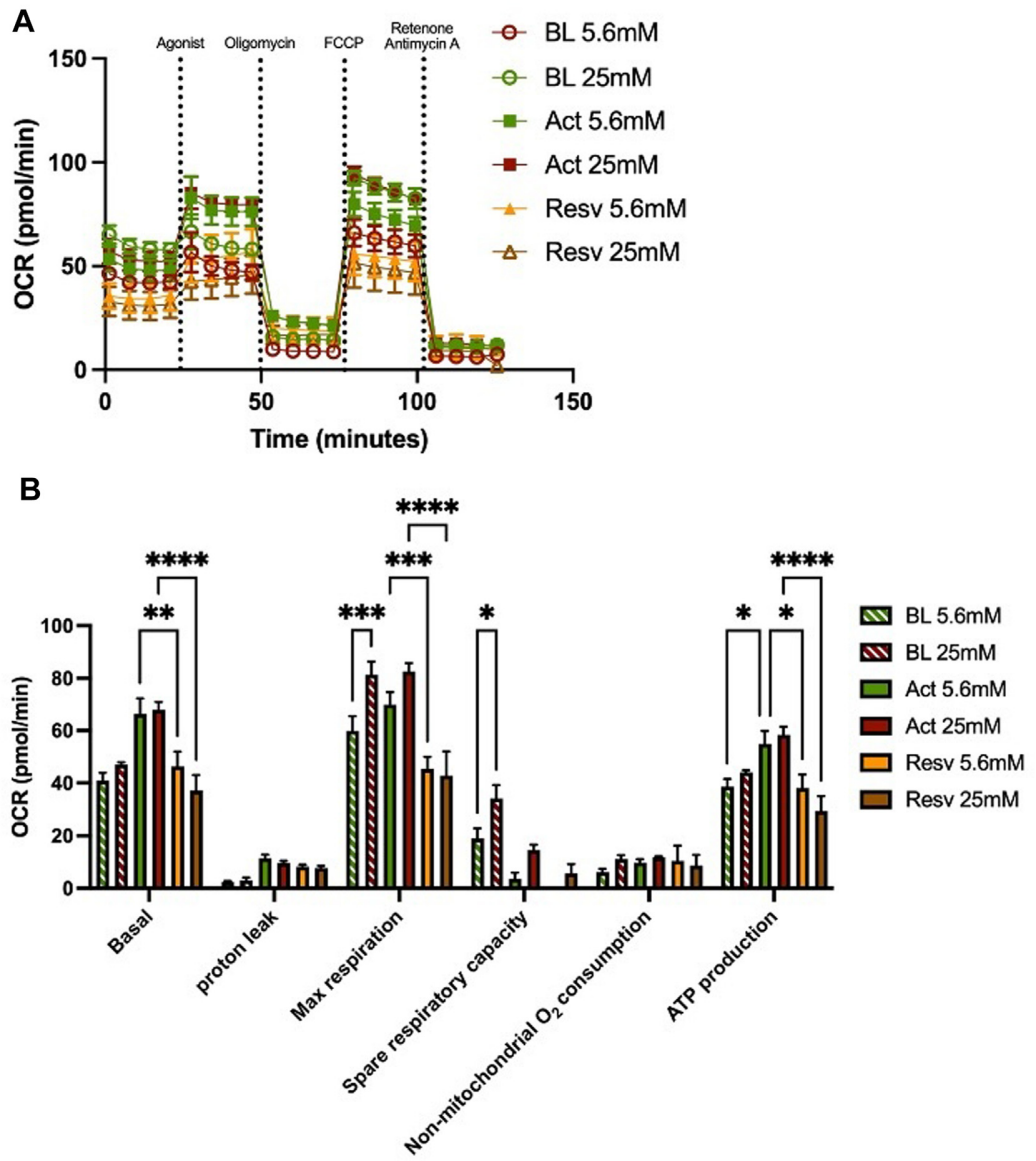
Quantification of oxygen consumption rate (OCR) in normoglycemia and acute hyperglycemia in the presence of resveratrol (Resv). Mitochondrial stress test was performed on healthy volunteers’ isolated platelets in the presence and absence of Resv using Seahorse XF Analyzer to determine OCR. Healthy volunteers’ platelets were isolated in isolation media containing 5.6 mM vs 25 mM glucose in isolation buffer (representing normoglycemia and acute hyperglycemia, respectively). Isolated platelets were treated with Resv (20 µM) for 45 to 60 minutes prior to activation and analysis. (A) Once baseline (BL) OCR levels were determined, platelets were activated (Act) with 100 ng/mL convulxin, followed by subsequent injections of oligomycin [adenosine triphosphate (ATP) synthase inhibitor], carbonylcyanide-p-trifluoromethoxyphenylhydrazone (uncoupling agent), and rotenone and antimycin A (complex I and III inhibitors) to determine OCR profile. Agonist was not added to BL samples. From the OCR curve, (B) basal respiration, proton leak, maximal respiration, spare respiratory capacity, nonmitochondrial oxygen consumption, and ATP production were determined. Results are shown as mean ± SEM, *n* = 3 to 4. ∗*P* < .05; ∗∗*P* < .01; ∗∗∗*P* < .001; ∗∗∗∗*P* < .0001 difference.

As resveratrol had a more pronounced effect on ROS under acute hyperglycemic conditions, in addition to a substantial decrease in mitochondrial calcium flux under the same condition, this compound was selected for mitochondrial respiration testing. Platelets from healthy volunteers were isolated under normoglycemic or hyperglycemic conditions and incubated with resveratrol or equivalent DMSO concentration (control). BL OCRs were determined for all conditions prior to activation of platelets. From the OCR profile (Figure 3A), specific parameters were determined (Figure 3B).

Basal maximal respiration and spare respiratory capacity (basal respiration subtracted from maximal respiration) were increased (26% ± 7% and 45% ± 11%, respectively; *P* < .05) in acute hyperglycemic conditions compared with normoglycemia control. A significant increase in ATP production after activation in normoglycemia was observed compared with BL (30% ± 5%; *P* < .05). Resveratrol decreased basal respiration (after activation) in normoglycemia and acute hyperglycemia conditions (30% ± 8% and 45% ± 8%, respectively; *P* < .01). Maximal respiration after activation was also decreased by resveratrol under both conditions (35% ± 7% and 48% ± 11% for normoglycemia and hyperglycemia, respectively; *P* < .001). In addition, ATP production under normoglycemia and acute hyperglycemia conditions was reduced by resveratrol (30% ± 9% and 49% ± 10%, respectively; *P* < .05). No significant changes were observed for proton leak (basal respiration not coupled to ATP synthesis) or nonmitochondrial oxygen consumption for the conditions tested (Figure 3B).

### 3.6 Resveratrol modulates procoagulant platelet number

**Figure 4:**
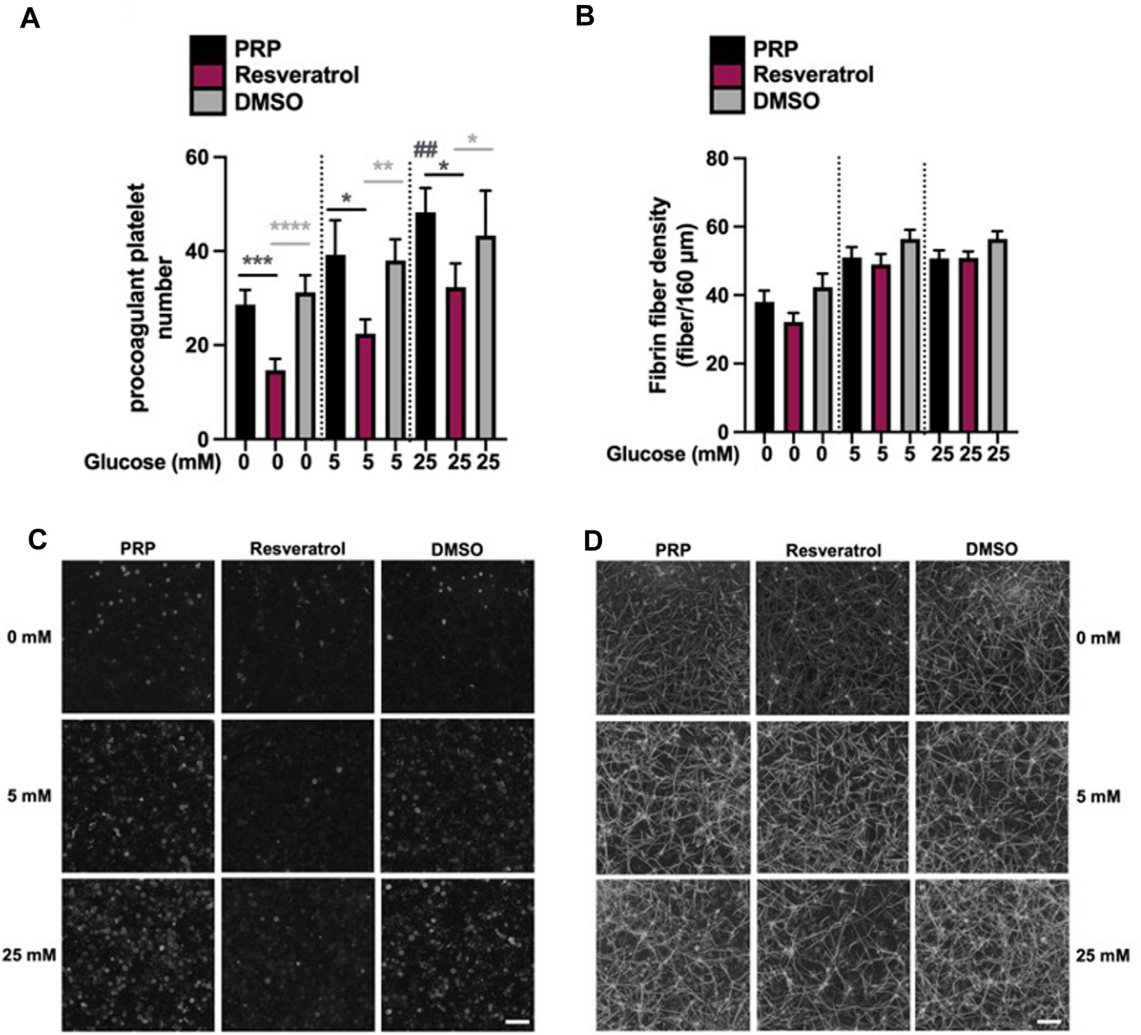
Procoagulant platelet and fibrin fiber quantification in clots formed in the presence of resveratrol in normoglycemic vs acute hyperglycemic conditions. Healthy volunteers’ platelet-rich plasma (PRP) was diluted with saline containing no glucose, 5 mM (representative of normoglycemia), or 25 mM (representative of hyperglycemia) glucose. (A) Absolute procoagulant platelet number was determined by laser scanning confocal microscopy. (B) Fibrin fiber density was determined by laser scanning confocal microscopy. (C) Representative confocal images of procoagulant platelets (annexin V). (D) Representative confocal images of fibrin fibers (Alexa Fluor-488 fibrinogen). “PRP” refers to clots formed in the absence of resveratrol, and “Resveratrol” refers to clots formed in the presence of 20 µM of the compound. Dimethyl sulfoxide (“DMSO”) refers to clots formed in the presence of an equivalent concentration of this solvent in the “Resveratrol” sample as a control. Tissue factor was used to initiate clotting following 20-minute incubation with 0 to 5 mM glucose ± resveratrol. Results are shown as mean ± SEM, *n* = 4. ∗*P* < .05; ∗∗*P* < .01; ∗∗∗*P* < .001; ∗∗∗∗*P* < .0001 difference from PRP control or DMSO in black and gray, respectively. ^##^*P* < .01 difference from 0 mM PRP control. Scale bar represents 25 µm.

Platelet mitochondrial respiration has been suggested to contribute to the development of procoagulant platelets [14,15]. Therefore, we investigated the effects of resveratrol on procoagulant platelet development, followed by clotting of PRP triggered with tissue factor. Acute hyperglycemia (dilution and incubation of PRP with 25 mM glucose) significantly increased procoagulant platelet number compared with saline control (48 ± 5 vs 29 ± 3, respectively; *P* < .01; Figure 4A). Quantification of procoagulant platelets (using annexin V) was performed from laser scanning confocal images (Figure 4C). Procoagulant platelet number was reduced following resveratrol treatment in saline control (15 ± 3 for resveratrol treatment; *P* < .001) and in normoglycemic (22 ± 3 and 39 ± 7 for resveratrol treatment and control, respectively; *P* < .05) and hyperglycemic conditions (32 ± 5 for resveratrol treatment; *P* < .05; Figure 4A).

### 3.7 Thrombus formation and clot structure measurements

**Figure 5:**
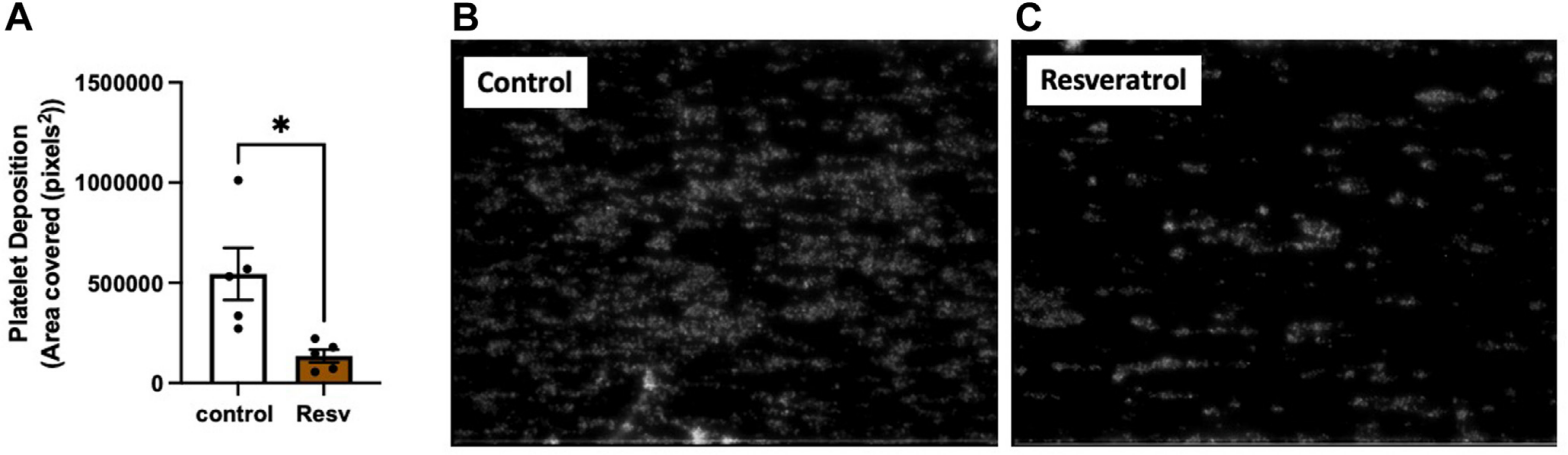
Thrombus formation under shear flow in normoglycemia in the presence of resveratrol (Resv). Healthy volunteers’ platelets were isolated in isolation media containing 5 mM glucose (representing normoglycemia). Isolated platelets were treated with Resv (20 µM) or buffer (control) and stained with 0.5 mM DIOC6 for 15 minutes. Washed red blood cells were added to samples to a final hematocrit of 40%. Red blood cell–platelet mixture was perfused over 20 μg/mL of a collagen-coated chip using a microfluidic syringe pump and recorded for 5 minutes at 1500 s^−1^. (A) Platelet deposition at 5-minute time point from when platelets first adhered to collagen strands was quantified from images. Representative image of platelet deposition in (B) control and (C) Resveratrol treated platelets. Results are shown as mean ± SEM, *n* = 5. ∗*P* < .05 difference from control.

**Figure 6:**
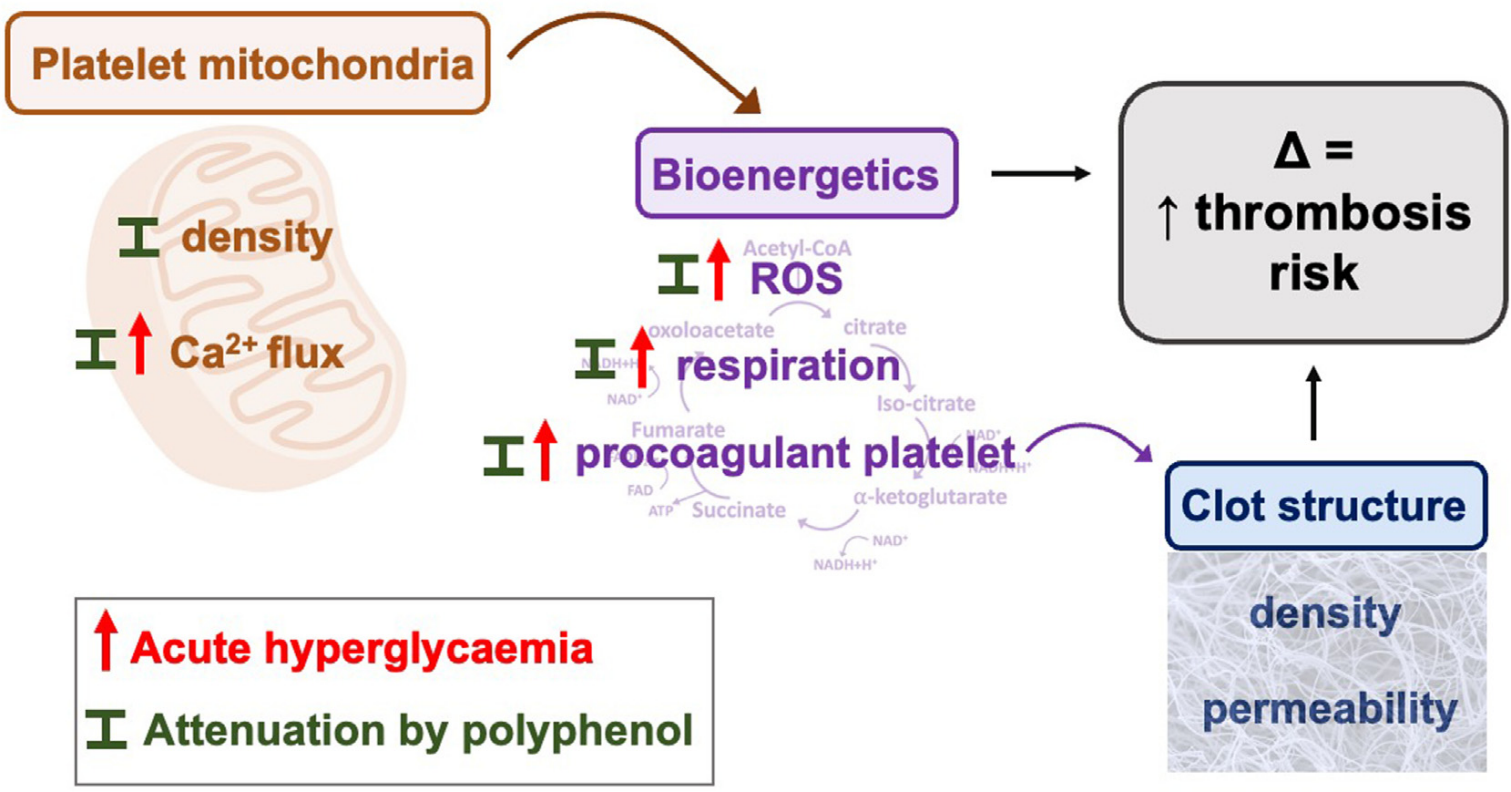
Summary schematic of the relationship between platelet bioenergetics and clot structure with thrombosis risk. Platelet mitochondrial (brown) is a central regulator of reactive oxygen species (ROS), energy consumption and expenditure (respiration), and procoagulant platelet formation. Platelet mitochondrial features that may influence bioenergetics (purple) include mitochondrial number (density) and calcium (Ca^2+^) influx. Procoagulant platelets support coagulation, having an impact on clot structure (blue) phenotype (eg, clot density and permeability). Altered (Δ) platelet bioenergetics and clot structure increase (↑) thrombosis risk. Red upward arrow represents observed increase with acute hyperglycemia, and I represents attenuation effect by polyphenols. ATP, adnosine triphosphate; CoA, coenzyme A; FAD, Flavin Adenine Dinucleotide; NAD, nicotinamide adenine dinucleotide; NADH, nicotinamide adenine dinucleotide hydrogen.

Conversely to the effects of resveratrol on procoagulant platelets discussed above, we observed no effect of this compound on fibrin fiber density in clots made with PRP under the conditions tested (Figure 4B). Fibrin fiber density (Alexa Fluor-488 fibrinogen) was determined from laser scanning confocal images (overlayed with procoagulant platelet channel; Figure 4D). We further observed no significant changes in clot pore size, indicative of clot architecture, in clots treated with resveratrol and other polyphenols under normoglycemic and acute hyperglycemic conditions (Supplementary Figure S2). There were no differences in PRP maximal clot firmness or clotting time in thromboelastic analysis of the extrinsic and intrinsic pathways in the presence of polyphenols (Supplementary Figure S3A, B). The kinetic profile of fibrin polymerization in PRP clots was also unchanged in the presence of polyphenols (Supplementary Figure S4A–C). Thrombus formation, measured under arterial shear, demonstrated a significant decrease in platelet deposition under normoglycemic conditions in the presence of resveratrol (75% ± 5% reduction with resveratrol treatment; *P* < .05; Figure 5A–C; Supplementary Videos S1 and S2). A summary schematic of our main findings is presented in Figure 6.

## 4 DISCUSSION

Our study shows that polyphenols attenuate increased ROS and energy consumption brought about by acute hyperglycemia in platelets. A summary of results is shown in Table 2. We also demonstrated that platelet procoagulant number, elevated in acute hyperglycemia, is reduced in the presence of resveratrol. Oxidative stress and increased ROS in hyperglycemia have been associated with pathogenesis of diabetes and other metabolic diseases [38]. Polyphenols have well-characterized antioxidant properties and recent meta-analyses of randomized controlled trials and population-based cohort studies show that polyphenols reduce cardiometabolic and cardiovascular disease mortality [[22], [23], [24]]. Previous studies have shown that polyphenols modulate postprandial glycemic response *in vivo* and decrease hyperglycemic-induced oxidative stress *in vitro* [[25], [26], [27]]. In this study, we also observed increased ROS and OCRs in hyperglycemia, consistent with previous reports [16,39], and newly show that mitochondrial calcium flux increases in platelets under hyperglycemic conditions. We also explore, for the first time, the effect of polyphenols on platelet bioenergetics and clot structure in the context of hyperglycemia.

**Table 2:** Summary of effects of normoglycemia vs acute hyperglycemia ± polyphenols on platelet bioenergetics and clot structure.

Parameter measured	Normoglycemic conditions + polyphenols	Acute hyperglycemic conditions	Acute hyperglycemic conditions + polyphenols
ROS (basal)	↓ with resveratrol, hesperetin, quercetin, and EGCG	↑	↓ with resveratrol, quercetin, and EGCG
Mitochondrial density (basal)	↓ with quercetin and EGCG	ns	ns
Mitochondrial Ca^2+^ flux	ns	↑	↓ with resveratrol and EGCG
Mitochondrial stress test (resveratrol)	↓ basal resp. after activation ↓ max resp. after activation ↓ ATP product. after activation	↑ BL max resp. ↑ BL spare resp. capacity	↓ basal resp. after activation ↓ max resp. after activation ↓ ATP product. after activation
Procoagulant platelets (resveratrol)	↓	↑	↓
Thrombus formation under shear flow (resveratrol)	-	↓	-
Fiber density	ns	ns	ns
Pore size	ns	ns	ns

**“**↑” indicates significant increase, “↓” indicates significant decrease.

ATP, adenosine triphosphate; BL, baseline; resp., respiration; EGCG, epigallocatechin gallate; ns, nonsignificant change; ROS, reactive oxygen species.

We observed that polyphenols decrease platelet ROS in both normoglycemic and acute hyperglycemic conditions. Interestingly, resveratrol had a more pronounced effect in the acute hyperglycemic condition. As mitochondria are central regulators of ROS formation [40,41], we explored the effect of hyperglycemia and treatment of polyphenols on 1) mitochondrial density, 2) mitochondrial calcium flux, and 3) OCR. Polyphenols decreased mitochondrial density in platelets under normoglycemia, consistent with previous reports on a liver cell line [27]. It has been hypothesized that changes in mitochondrial density by polyphenols (quercetin) are attributable to lowered lipogenesis [27]. Recent studies have demonstrated the crucial role of *de novo* lipogenesis in the differentiation of megakaryocytes and platelet production [42,43]. Further characterization is required to determine the mechanism via which polyphenols contribute to decreased mitochondrial density in platelets. In this study, we observed no significant changes in mitochondrial citrate synthase activity in hyperglycemia in the absence or presence of polyphenols. This suggests that the increased ROS in hyperglycemia is unlikely due to elevated mitochondria density. Therefore, we investigated the impact of hyperglycemia on mitochondrial calcium flux. Acute hyperglycemia increased platelet mitochondrial flux, indicating elevated metabolism. Two compounds, namely resveratrol and EGCG, attenuated mitochondrial calcium flux. This suggests that polyphenols have the potential to modulate platelet energy expenditure/production, contributing to more stable glycemic control. Our findings are consistent with previous reports of improved mitochondrial bioenergetics by polyphenols in pancreatic beta-cells and neuron cultures [44,45], proposing benefits of compounds in the context of diabetes and inflammation.

It has been reported that platelets in individuals with diabetes are “hyperactive” and that calcium flux influences mitochondrial respiratory capacity and function [9,46]. Therefore, to establish how changes in mitochondrial calcium flux impacted platelet metabolism, we explored the effect of hyperglycemia and resveratrol on OCR. As expected, we observed increased respiration in acute hyperglycemic conditions, while platelet activation (P-selectin and PAC1 in response to varying concentrations of convulxin) remained unchanged in comparison with normoglycemia (data not shown). Resveratrol modulated OCR in both normoglycemic and acute hyperglycemic conditions. This suggests that this compound may have potential benefits in attenuating oxidative metabolism, thereby contributing to improved glycemic control and decreased inflammation [47,48].

Platelet energy metabolism and mitochondrial respiration have been linked with development of procoagulant platelets [14,15]. Furthermore, calcium mobilization, followed by water influx, gives procoagulant platelets their distinctive “balloon”-like morphology [49]. A procoagulant state has been reported in patients with diabetes, including changes in platelet function and activation of the coagulation cascade [10,11] and increased procoagulant platelet priming in hyperglycemia [16]. Therefore, we explored the effect of acute hyperglycemia and resveratrol treatment on procoagulant platelet formation and clot structure. We observed that acute hyperglycemia increased procoagulant platelets, as expected, and that resveratrol decreased procoagulant platelet numbers in normoglycemic and hyperglycemic conditions. This is in agreement with the mitochondrial calcium flux and stress test results and indicates that resveratrol may contribute to decreased prothrombotic state. Nevertheless, we observed no significant effects of acute hyperglycemia on fiber density or clot porosity. Furthermore, polyphenols tested in this study did not alter fiber density, clot pore size, clot firmness in extrinsic/intrinsic pathway-specific analyses, or clot polymerization. The reason for this discrepancy between increased procoagulant platelet formation and an absence of the effect on clot structure is currently unknown. It may be that the PRP clot structure and function tests are not sensitive enough for changes in platelet procoagulation. Alternatively, there may be other mechanisms at play or contributing factors present *in vivo* that attenuate the effects of procoagulant platelets under the conditions tested in our clot structure assays. We hypothesize that the effects of hyperglycemia and protection by polyphenols (namely, resveratrol) on ROS are related to changes in OCR. Further investigations are required to determine if and/or how these changes contribute to procoagulant platelet development.

Although there are some discrepancies [[50], [51], [52]], previous reports have suggested a role for polyphenols/extracts as antithrombotic agents, including decreased platelet aggregation by resveratrol [31,32]. Resveratrol has also been shown to decrease collagen-induced thrombus formation in samples from patients with diabetes compared with control [32]. In our hands, this compound also decreased platelet/PRP aggregation (data not shown) and platelet deposition under shear flow. These reports support our findings that polyphenols may have beneficial effects on platelet bioenergetics in hyperglycemia and, thereby, attenuate elevated risk of thrombosis in diabetes. Further investigations *in vivo* are required to establish the effect of chronic hyperglycemia on platelet bioenergetics and the potential benefits of polyphenols on thrombosis risk.

Unlike previous reports of changes in turbidity and clot firmness by food extracts [51,53,54], we did not observe an effect of the polyphenols tested on clot structure. It is possible that other compounds present in specific food extracts included in these studies, single or in combination, may influence clot structure. Differences in incubation times, dosage, and sample preparation (eg, whole blood vs PRP) may also contribute to differences in observations. More recently, a study showed prolonged occlusion time in a ferric-chloride injury model following treatment with flavonoid luteolin but no changes in prothrombin or partial thromboplastin time [52]. Further investigations are required to establish the effect of purified polyphenols and/or food extracts on clot structure and thrombus formation/stability. The presence of multiple hydroxyl groups contributes to the free radical scavenger potential of the compounds used in this study [55,56]. As shown in Table 1, resveratrol is the only compound that does not share the common double-ring structure of compounds from the flavonoid class. Structural characteristics of each compound may contribute to their different effects on ROS attenuation and platelet bioenergetics. Further studies are required to identify the mechanisms of action associated with specific compounds/classes. Furthermore, the effect of compounds on platelet bioenergetics in acute hyperglycemia presented in this study supports the need for future studies to determine the potential chronic effects of compounds on hyperglycemia.

Our study explores for the first time the effect of polyphenols on platelet bioenergetics in normoglycemia vs acute hyperglycemia and the subsequent effect on clot structure *in vitro*. We show that compounds tested attenuated increased platelet bioenergetics and metabolism stemming from acute hyperglycemia. Our data suggest that polyphenols may influence prothrombotic phenotype by affecting cellular function rather than clot structure. In conclusion, polyphenols may have potential benefits at attenuating platelet-mediated elevated thrombosis risk in diabetes and may therefore potentially contribute to thrombosis prevention strategies.
