# Supplementary material for: Plant-derived compounds normalize platelet bioenergetics and function in hyperglycemia
Source: Res Pract Thromb Haemost. 2024 Aug 14;8(6):102548. doi: 10.1016/j.rpth.2024.102548 (PMC11416496; doi:10.1016/j.rpth.2024.102548)
Supplement: Supplementary Material [file mmc3.docx]

**Plant-derived compounds normalise platelet function in hyperglycaemia**

Julia S. Gauer^1^*, Abigail Ajanel^2^, Lutale M. Kaselampao^1^, Isabel Candir^1^, Amanda D. V. MacCannell^1^, Lee D. Roberts^1^, Robert A. Campbell^2,3^, Robert A. S. Ariëns^1^.

Supplementary material

**SUPPLEMENTARY METHODS**

**Mitochondria-derived ROS**

Platelets from healthy volunteers were isolated with modified Tyrode’s buffer containing 5.6mM vs 25mM glucose to represent normo- and acute hyperglycaemia, respectively. Washed platelets were labelled with platelet marker CD41-APC (559777, BD) and mitochondria-derived ROS marker mitoSOX (M36008) for 15mins at 37°C away from light in the presence and absence of polyphenols (20 μM). Basal platelet mitochondria-derived ROS was measured with CytoFLEX S Flow Cytometer (Beckman Coulter).

**Clot permeation**

Analysis of clot permeability was performed as previously described[1]. Briefly, platelet-rich plasma (PRP) was diluted 1:6 with saline (154mM NaCl) containing 0mM, 5mM (representative of normoglycaemia) or 25mM glucose (representative of hyperglycaemia). Polyphenols (20μM) were added to samples and incubated for 20min at RT. Clotting was initiated with addition of tissue factor (1pM) and CaCl_2_ (10mM) and sample was immediately transferred to Ibidi uncoated μ-Slide 0.4mm (Ibidi GmbH) then placed in a humidity chamber for 1hr. A plastic syringe was attached to each well containing a clot and filled to the same height (4cm) to apply constant pressure. Rate of buffer flow through the clot was determined by plotting flow-through volume (correlated to weight, assuming 1g=1mL) over time, fitted by linear regression (R^2^≥0.99). The permeation coefficient (Ks; Darcy constant) was obtained as previously described[2].

**Rotational thromboelastometry**

Thromboelatometric analysis was performed using diluted PRP (1:6) on a ROTEM-Delta (Werfen). PRP was incubated with polyphenols (20μM) for 20mins at RT prior to analysis. Tests were initiated with CaCl_2_ and tissue factor (EXTEM) or phospholipid and ellagic acid (INTEM) for analysis of the extrinsic and intrinsic pathways, respectively. Clotting time (CT) and maximum clot firmness (MCF) was obtained.

**Kinetic profile of polymerizing fibrin clots**

The kinetic profile of polymerizing fibrin clots was investigated using methods previously described[3]. In brief, PRP was diluted 1:6 with saline. Samples were incubated with polyphenols (20μM) for 20mins at RT and transferred to half-volume 96-well plate (Thermo Fisher), in triplicates. Clotting was initiated with CaCl_2_ (5mM) and tissue factor (1pM), and changes in absorbance (optical density) were measured at 340nm every 17s for 1hr at 37°C using a Powerwave microtiter-plate reader (Bio-Tek). From turbidity profile, average rate of clotting (corresponding to rate between 25-75% clotting) and maximum absorbance (indicative of fibre diameter) were obtained.

**SUPPLEMENTARY FIGURES**

**
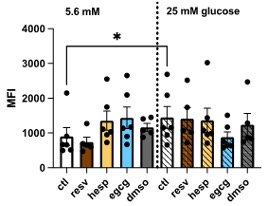
**

**Supplementary Figure S1 Mitochondria-derived ROS in the presence of polyphenols in normo- vs hyperglycaemic condition.** Basal measure of mitochondria-derived ROS by flow cytometry (mitoSOX) in healthy volunteers’ platelets isolated in isolation media containing 5.6mM vs 25mM glucose in isolation buffer (representing normo- and acute hyperglycaemia, respectively) in the presence and absence of 20 μM polyphenols. Results shown as mean ± s.e.m, n=5-6. *p<0.05 difference.

**
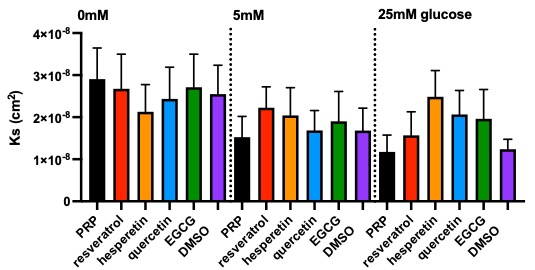
**

**Supplementary Figure S2 Porosity of clots formed in the presence of polyphenols in normo- vs acute hyperglycaemic conditions.** Healthy volunteers’ platelet-rich plasma (PRP) was diluted with saline containing no glucose, 5mM (representative of normoglycaemia) or 25mM (representative of hyperglycaemia) glucose. Tissue factor was used to initiate clotting following 20min incubation with 0-5 mM glucose ± 20μM polyphenols. Porosity PRP clots was determined by the permeation coefficient (K_s_). Higher K_s_ values are indicative of a more porous fibrin fibre network, and lower K_s_ values of a less porous fibrin fibre network. ‘PRP’ refers to clots formed in the absence of polyphenols and ‘DMSO’ refers to clots formed in the presence of equivalent concentration of this solvent, as a control. Results shown as mean ± s.e.m, n=4.

**
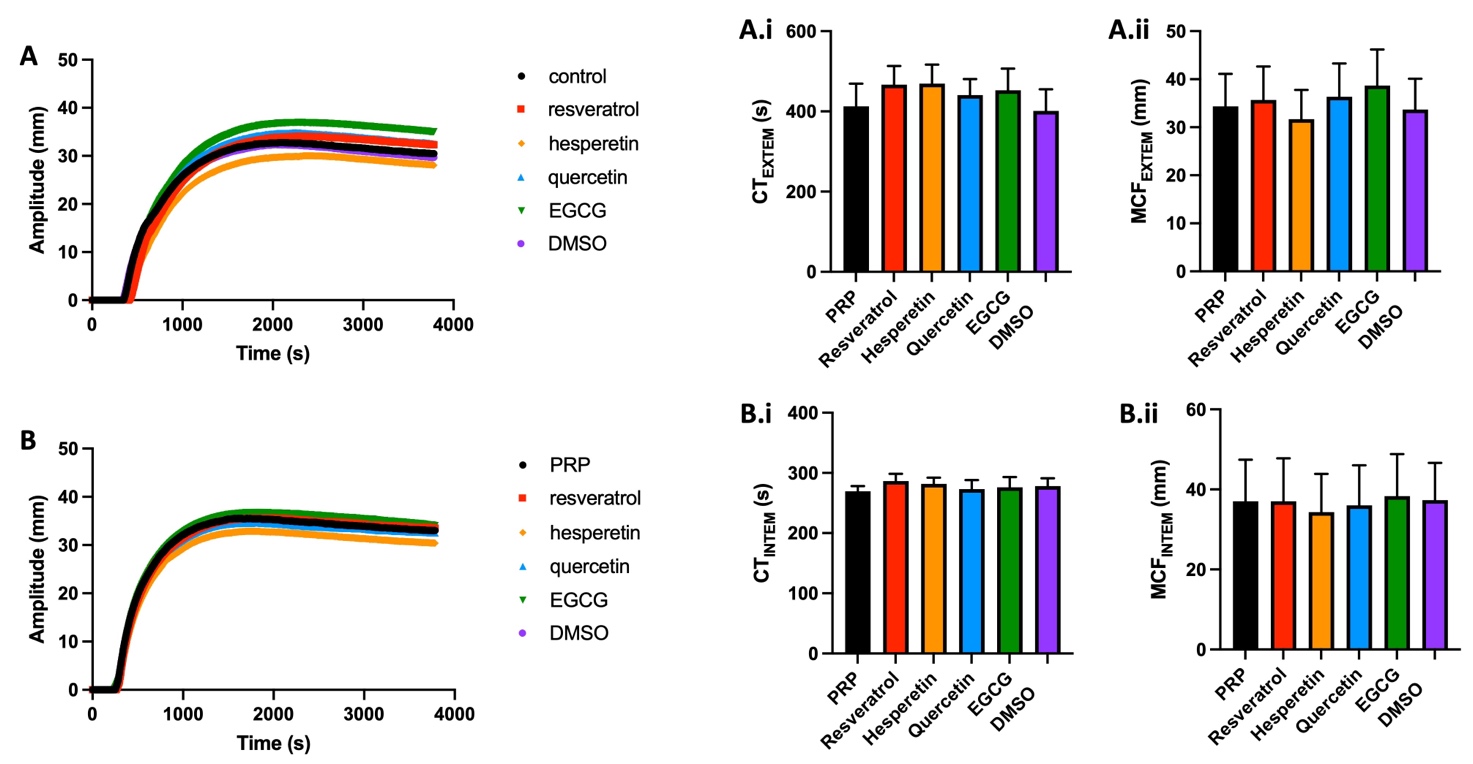
**

**Supplementary Figure S3 Thromboelastometric analysis of clots formed in the presence of polyphenols.** Healthy volunteers’ platelet-rich plasma (PRP) was diluted with saline and incubated with polyphenols (20μM) for 20mins at RT. Analysis of the extrinsic (EXTEM; **A**) and intrinsic pathways (INTEM; **B**) were performed, producing a curve of changes in amplitude over time. From this curve, clotting time (CT; **i**) and maximum clot firmness (MCF; **ii**) were obtained. Results shown as mean ± s.e.m, n=3.

**
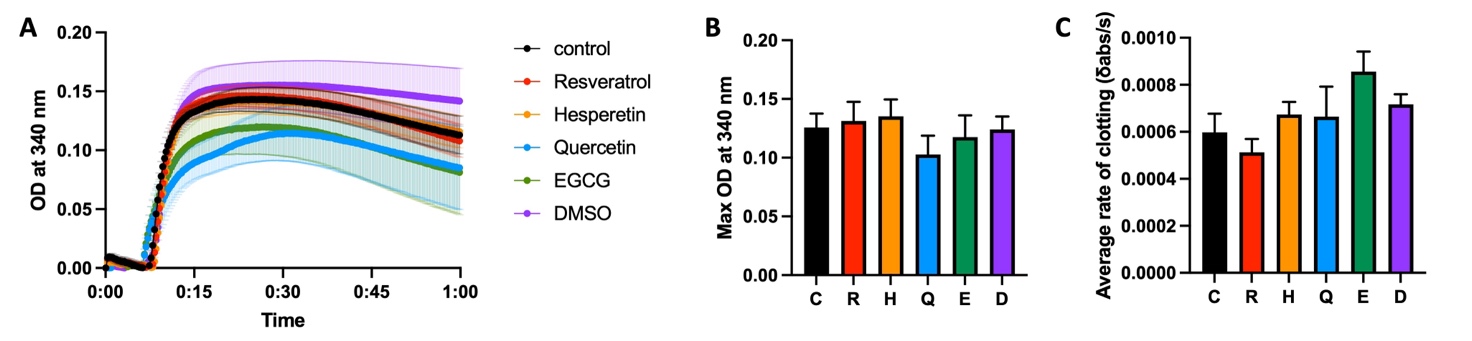
**

**Supplementary Figure S4 Turbidity analysis of polymerizing clots in the presence of polyphenols.** Healthy volunteers’ platelet-rich plasma (PRP) was diluted with saline and incubated with polyphenols (20μM) for 20mins at RT. From the kinetic profile of polymerizing clots (**A**), maximum absorbance (**B**) and average rate of clotting (**C**) were obtained. Results shown as mean ± s.e.m, n=4-6.

[**Control Platelets**](https://leeds365-my.sharepoint.com/:v:/g/personal/medjsanb_leeds_ac_uk/EV7BuUW4pMVCsPH_fpbBOVAB5eML1-2Nsy7e9zI4rX_C5g?nav=eyJyZWZlcnJhbEluZm8iOnsicmVmZXJyYWxBcHAiOiJPbmVEcml2ZUZvckJ1c2luZXNzIiwicmVmZXJyYWxBcHBQbGF0Zm9ybSI6IldlYiIsInJlZmVycmFsTW9kZSI6InZpZXciLCJyZWZlcnJhbFZpZXciOiJNeUZpbGVzTGlua0NvcHkifX0&e=Mtu9WF)

**Supplementary Video S1 Representative video of thrombus formation under shear flow in normo-glycaemia.**

[**Platelets treated with Resveratrol**](https://leeds365-my.sharepoint.com/:v:/g/personal/medjsanb_leeds_ac_uk/EdA4YdYAhF1ApSgiijMdWaEBgz4SjswakR8Y0a_FHSiblg?nav=eyJyZWZlcnJhbEluZm8iOnsicmVmZXJyYWxBcHAiOiJPbmVEcml2ZUZvckJ1c2luZXNzIiwicmVmZXJyYWxBcHBQbGF0Zm9ybSI6IldlYiIsInJlZmVycmFsTW9kZSI6InZpZXciLCJyZWZlcnJhbFZpZXciOiJNeUZpbGVzTGlua0NvcHkifX0&e=4cf8oF)

**Supplementary Video S2 Representative video of thrombus formation under shear flow in normo-glycaemia in the presence of 20μM** **resveratrol.**

**SUPPLEMENTARY REFERENCES**

1. Gauer JS, Duval C, Xu RG, Macrae F, McPherson HR, Tiede C, Tomlinson DC, Watson S, Ariens R. Fibrin-GPVI interaction increases platelet procoagulant activity and impacts clot structure. J Thromb Haemost. 2023;in press.

2. Pieters M, Undas A, Marchi R, De Maat MP, Weisel J, Ariens RA. An international study on the standardization of fibrin clot permeability measurement: methodological considerations and implications for healthy control values. J Thromb Haemost. 2012;10:2179-2181.

3. Gauer JS, Riva N, Page EM, Philippou H, Makris M, Gatt A, Ariëns RAS. Effect of anticoagulants on fibrin clot structure: A comparison between vitamin K antagonists and factor Xa inhibitors. Res Pract Thromb Haemost. 2020;4:1269-1281.
